# Biomarker‐guided clustering in an Amish population older than 60 reveals subgroups featuring distinct Alzheimer Disease pathologies

**DOI:** 10.1002/alz.089004

**Published:** 2025-01-09

**Authors:** Ping Wang, Yeunjoo E. Song, Audrey Lynn, Kristy L. Miskimen, Michael B. Prough, Daniel A. Dorfsman, Alex V. Gulyayev, Renee A. Laux, Sarada L. Fuzzell, Sherri D. Hochstetler, Andrew F Zaman, Larry D. Adams, Laura J. Caywood, Jason E. Clouse, Sharlene D. Herington, Patrice L. Whitehead, Yining Liu, Noel C. Moore, Leighanne R. Main, Paula K. Ogrocki, Alan J. Lerner, Anthony J. Griswold, Jeffery M. Vance, Michael L. Cuccaro, William K. Scott, Margaret A. Pericak‐Vance, Jonathan L. Haines

**Affiliations:** ^1^ Department of Population and Quantitative Health Sciences, Case Western Reserve University School of Medicine, Cleveland, OH USA; ^2^ Cleveland Institute for Computational Biology, Case Western Reserve University, Cleveland, OH USA; ^3^ John P. Hussman Institute for Human Genomics, University of Miami Miller School of Medicine, Miami, FL USA; ^4^ Dr. John T. Macdonald Foundation Department of Human Genetics, University of Miami Miller School of Medicine, Miami, FL USA; ^5^ John P. Hussman Institute for Human Genomics, University of Miami Miller School of Medicine, Miami, FL, USA, Miami, FL USA; ^6^ Department of Nutrition, Case Western Reserve University School of Medicine, Cleveland, OH USA; ^7^ Department of Genetics and Genome Sciences, Case Western Reserve University School of Medicine, Cleveland, OH USA; ^8^ Department of Neurology, Case Western Reserve University School of Medicine, Cleveland, OH USA; ^9^ Department of Neurology, University Hospitals Cleveland Medical Center, Cleveland, OH USA; ^10^ Case Western Reserve University School of Medicine, Cleveland, OH USA; ^11^ Department of Population and Quantitative Health Sciences, Case Western Reserve University, Cleveland, OH USA

## Abstract

**Background:**

Plasma amyloid‐beta (Aβ) 42/40 ratio and phosphorylated tau 181 (pTau181) are promising blood biomarkers for AD. Compared to heterogenous clinical phenotypes, they are more objective and proximal to the pathological hallmarks of Aβ plaques and tau tangles. Biomarker‐guided clustering using Aβ42/40 and pTau181 can potentially establish subpopulations that share similar mechanisms of AD and treatment responses.

**Method:**

Plasma biomarkers were measured using Simoa™ Neuro‐3Plex, 4Plex, and pTau181 Advantage V2 assays. To eliminate potential confounding effects, age, sex, and study center were regressed out of Aβ42/40 and pTau181 using a quadratic regression model. Adjusted biomarkers were then normalized for unsupervised K‐means clustering, with the optimal number of clusters determined by the elbow method and Silhouette score. To interpret the cluster profiles, we explored the differences in demographics and clinical features between clusters. We also examined the association between clusters and neuropsychological tests adjusting for age, sex, study center, APOE e4 alleles, and diagnosis.

**Result:**

We analyzed plasma biomarkers from 593 Amish individuals (age ³ 60 years old) living in Ohio and Indiana. 62% were females, and the average age was 81.7 ± 5.7 years. Utilizing plasma Aβ42/40 and pTau181, the clustering approach yielded six distinct clusters (N = 92, 95, 94, 152, 75, and 85) whose composition was driven by varying burdens of amyloid and tau pathology. There was no significant difference in proportion of cognitive impairment or of family structure across clusters. As expected, the low Aβ42/40 and high ptau cluster (Cluster 6) featured a significantly higher proportion of APOE e4 carriers than other clusters combined (51% vs 22%, p = 0.002). No significant associations were found between clusters and neuropsychological tests.

**Conclusion:**

The integrated analysis of plasma Aβ42/40 and pTau181 in the Amish established six clusters with varying levels of amyloid and tau burden, suggesting possible differences that need to be further explored. Examination of potential genetic or pathophysiological differences across the clusters are underway.

